# Feasibility, Safety, and Impact of the Probiotics Lactiplantibacillus plantarum and Bifidobacterium longum subspecies infantis in Papua New Guinean Infants: Protocol for a Randomized Controlled Trial

**DOI:** 10.2196/73926

**Published:** 2025-10-02

**Authors:** Rebecca L Ford, Andrew R Greenhill, Joycelyn Sapura, Amelia Koata, Mary Dreyam, Tilda Orami, Joe Jude, Madeline Ong, Wendy Kirarock, Dorcas Joseph, Geraldine Masiria, Celestine Aho, William S Pomat, Anita H J van den Biggelaar, Peter C Richmond

**Affiliations:** 1 Papua New Guinea Institute of Medical Research Goroka Papua New Guinea; 2 Microbiology Research Group Institute of Innovation, Science and Sustainability Federation University Churchill Australia; 3 Wesfarmers Centre for Vaccines and Infectious Diseases The Kids Research Institute Australia Perth Australia; 4 Centre for Child Health Research University of Western Australia Perth Australia; 5 Discipline of Paediatrics Medical School University of Western Australia Perth Australia

**Keywords:** probiotics, synbiotics, Bifidobacterium longum subspecies infantis, Lactiplantibacillus plantarum, infant health, neonatal infections, gut microbiota, colonization

## Abstract

**Background:**

Childhood mortality in low- or middle-income countries (LMICs) remains a major public health concern, with infections being a leading cause of infant death. Probiotics have shown promise in reducing infection-related morbidity and mortality in preterm infants, but their use in newborns born at or near term in LMICs requires further investigation.

**Objective:**

This study aims to assess the feasibility, safety, and initial outcomes of administering 1 of 2 synbiotic formulations or a placebo to newborns in the Eastern Highlands Province of Papua New Guinea.

**Methods:**

Following ethics approvals in 2018 and 2019, and receipt of the synbiotic preparations in September 2020, healthy neonates (<72 h old; n=244) were recruited between October 2020 and June 2023 and randomly assigned in a double-blind manner (1:1:1) to receive an oral preparation containing *Lactiplantibacillus plantarum*, *Bifidobacterium longum* subspecies *infantis*, or placebo for 7 consecutive days. Follow-up continued for 6 months, with rectal swabs, stool, blood, saliva, and nasopharyngeal swabs collected before the intervention; on day 7; at age 2 weeks; and at ages 1, 3, 4, and 6 months. Ongoing analyses will assess probiotic gut colonization, bacterial nasopharyngeal carriage, and antibody responses to routine childhood vaccines (*Hemophilus influenzae* type b; hepatitis B; 13-valent pneumococcal conjugate vaccine; and diphtheria, tetanus, and whole-cell pertussis) as well as hospitalization and infection rates among the intervention groups compared to the placebo group.

**Results:**

Recruitment began in October 2020, with the target sample size expanded from 195 to 240 in March 2023 owing to higher-than-anticipated loss to follow-up during the COVID-19 pandemic. Of the 244 enrolled infants, 218 (89.3%) completed the full 7-day synbiotic or placebo course, and 169 (69.3%) completed the study. All follow-up visits concluded in December 2023. Disruptions due to the COVID-19 pandemic led to family relocations outside the study area, preventing some infants (75/244, 30.7%) from completing the study. High rates of sample collection were achieved, with rectal swabs (1452/1474, 98.1%), nasopharyngeal swabs (1255/1262, 99.44%), saliva samples (881/882, 99.9%), and blood samples (876/882, 99.3%) successfully obtained at multiple time points. Data analysis is ongoing and expected to be completed by the end of 2025.

**Conclusions:**

This study demonstrates that probiotic supplementation is feasible and safe in healthy infants in an LMIC such as Papua New Guinea. The findings from this study will inform the design of larger trials of probiotics and synbiotics to complement existing efforts to reduce infection-related infant mortality in LMICs, while also providing insights into their clinical, immunological, and microbiological impacts in infants.

**Trial Registration:**

Australian New Zealand Clinical Trials Registry (ANZCTR) ACTRN12620001369910; https://anzctr.org.au/Trial/Registration/TrialReview.aspx?ACTRN=12620001369910

**International Registered Report Identifier (IRRID):**

DERR1-10.2196/73926

## Introduction

### Childhood Mortality and Interventions

Childhood mortality remains one of the greatest public health challenges of our time. The global mortality rate for children aged <5 years was estimated at 38 per 1000 live births in 2021, which equates to an estimated 5 million deaths per year in this age group [1]. The mortality rate has decreased over the past 30 years, with the greatest improvements observed in children aged 1 month to 5 years. However, neonatal deaths (within the first 28 d of life) accounted for almost half of all deaths of children aged <5 years globally in 2022, highlighting the disproportionate mortality burden in the neonatal period [2].

The majority of childhood deaths occur in low- or middle-income countries (LMICs). Poor sanitation, limited access to improved water, poor access to health care, limited childhood vaccination coverage, inadequate nutrition, and the cost of health care relative to income contribute to high infectious disease burden and poor health outcomes. Many of these long-recognized contributors are difficult to rectify in the short term. To expedite improvements in child health in LMICs, novel approaches are required to complement longer-term initiatives.

### Probiotics and Synbiotics

Probiotics have long been ingested by humans through the consumption of fermented foods. The World Health Organization defines probiotics as “live microorganisms which when administered in adequate amounts confer a health benefit on the host” [3]. In 2013, a panel of experts supported the ongoing use of this definition [4,5]. In addition to fermented foods, probiotics have been isolated from various sources and commercialized as health supplements, and, more recently, as therapeutic agents. A promising strategy for the use of probiotics is in combination with prebiotics: nondigestible food ingredients that selectively promote microbial growth. Termed “synbiotics,” these combinations are thought to be more effective in enhancing the survival and activity of the probiotic bacteria once ingested and have the potential to lower the risk of developing many illnesses and diseases [6,7]. However, the quality of synbiotic products available on the market varies significantly, with differences in probiotic strains, prebiotic types, and dosage levels that may affect their effectiveness [8]. Furthermore, while initial studies show promise [9-11], more robust clinical trials are needed to confirm their therapeutic benefits and safety because much of the current evidence is based on in vitro or animal models [12-14], rather than large-scale human studies. Standardization in product formulation and more rigorous clinical proof are essential to fully understand their potential health impacts.

### Study Rationale and Objectives

One novel approach that has garnered interest in recent years is the use of probiotics early in life. In particular, probiotics have been shown to improve health outcomes in preterm infants. Meta-analyses of the health outcomes of probiotics administration in preterm infants demonstrate that probiotics can decrease the risk of necrotizing enterocolitis (NEC) [15-17] and death [18]. The administration of probiotics to preterm infants is now often standard practice in neonatal intensive care units in many high-income settings. Its applicability has been investigated in some LMICs for at least 20 years [19], with a systematic review of randomized controlled trials conducted in India suggesting that probiotics reduce the risk of NEC, late-onset sepsis, and death [20]. Similarly, another review of trials conducted in 10 LMICs demonstrated that probiotics decreased the risk of NEC, late-onset sepsis, and all-cause mortality [21]. Nevertheless, in 2023, the US Food and Drug Administration raised safety concerns regarding the use of probiotics in preterm infants in hospitals [22]. Unlike medical products, probiotics used in neonatal wards have not undergone the agency’s premarket evaluation of safety, effectiveness, and quality for medical use**.** Given the potential for harm posed by products in individuals considered highly vulnerable, such as preterm infants, the Food and Drug Administration has urged industry stakeholders as well as clinical and research funding communities to focus on high-quality clinical trials using products that meet quality criteria to provide definitive evidence to inform the use of these products by health care providers.

There has been interest over the past decade in the potential role of probiotics in improving health in full-term infants in LMICs. A large community-based study in India has demonstrated the potential health benefits of probiotics in infants [23]. In a randomized controlled trial, >2000 children in the intervention arm were administered a strain of *Lactobacillus plantarum—*currently classified as *Lactiplantibacillus plantarum*—supplemented with fructo-oligosaccharide (together forming a synbiotic) once daily over 7 days, starting on days 2 to 4 of life. Compared to the placebo group, children in the synbiotic intervention arm had significantly lower rates of a composite of sepsis (including culture-positive sepsis, culture-negative sepsis, and lower respiratory tract infections [LRTIs]) or death (the study’s primary outcome). Reductions were also observed in individual components of sepsis, including LRTIs, which constituted the majority of cases. In addition, the intervention group experienced lower rates of diarrhea, omphalitis, and other local infections.

Papua New Guinea (PNG) is an LMIC in which approximately 40% of the population live in poverty [24]. In PNG, approximately 4.1% of children die before reaching the age of 5 years, and the mortality rate for children aged <1 year is approximately 33 deaths per 1000 live births, which exceeds global averages [25]. The leading causes of death in children in PNG include neonatal complications (such as preterm birth, birth asphyxia and birth trauma, and sepsis), LRTIs, tuberculosis, malaria, and diarrhea. The current neonatal mortality rate alone is estimated to be 9.25% [26]. LRTIs, often caused by pneumococcal infections, play a particularly critical role in child morbidity and mortality in PNG. The high diversity and prevalence of nasopharyngeal pneumococcal carriage in children, compounded by limited vaccine coverage, underscore the importance of understanding local serotype distribution and the effectiveness of 13-valent pneumococcal conjugate vaccine (PCV13) antibodies in this setting [27,28]. Given the high burden of serious infections in children in PNG, there is a need to explore novel approaches to reducing this burden.

In this study, we report on the implementation and participant recruitment of a double-blind, randomized, placebo-controlled trial conducted in the Eastern Highlands Province (EHP) of PNG, in which newborns were administered 1 of 2 synbiotic formulations—*L plantarum* (American Type Culture Collection [ATCC] 202195) with fructo-oligosaccharide or *Bifidobacterium longum* subspecies *infantis* (Bi-26) with human milk oligosaccharide—or a placebo. This was the first study of probiotics supplementation in infants in PNG; therefore, the primary aim of this pilot study was to demonstrate the feasibility and safety of giving infants in PNG probiotic supplementation for 7 consecutive days, starting within the first 72 hours of life, before embarking on a larger-sized clinical impact trial. The secondary and exploratory aims include assessing gut colonization by probiotic species at multiple time points, hospitalization and infection rates, nasopharyngeal carriage of respiratory bacteria, vaccine-specific immune responses, microbiome development, and maternal vaginal colonization at delivery.

## Methods

### Study Location

PNG is one of the largest countries in the Oceania region by both land area and population. There has been rapid population growth over the past 30 years, from <4 million in 1990 to an estimated 11.8 million in 2021 [29,30].

This study was conducted in the EHP of PNG, where an estimated 785,000 people live. The capital of the EHP is Goroka, with the town (with a population of approximately 33,000) and surrounding periurban areas having a total population of approximately 212,000 [30]. Goroka is located approximately 1600 meters above sea level and is situated on the Highlands Highway (the main highway in PNG). The climate is typically highland subtropical: rainfall occurs year round, with reduced precipitation from June to September. The terrain is mountainous and rugged, making access to many rural villages and communities outside of town challenging and requiring off-road 4-wheel-drive vehicles.

The study was conducted by the PNG Institute of Medical Research (PNGIMR) and international collaborators. The PNGIMR is headquartered in Goroka. Participants were recruited at the Goroka Provincial Hospital, the major referral hospital for the EHP.

### Study Design and Objectives

In this pilot study, we aimed to evaluate the administration of 2 different synbiotic formulations compared to placebo in newborns in PNG during the first week of life. The study design was a double-blind, randomized, placebo-controlled trial in which newborns received 1 of 2 synbiotic formulations or a placebo over 7 consecutive days, with the first dose given before the newborn was aged 72 hours and administration completed within the first 10 days of life. The overall objective is to generate data that will inform the design and implementation of a larger, definitive clinical trial in infants in PNG, including the assessment of clinical end points.

The primary pilot study objectives were (1) safety in terms of intolerance (diarrhea, vomiting, abdominal distension, and weight gain) or probiotic sepsis during the 7-day supplementation period, leading to the cessation of the study medication; and (2) the feasibility and cultural acceptability of daily probiotic administration (the proportion of children who received all 7 daily doses of supplementation without missing any). The secondary objective was to demonstrate the colonization of the gut by probiotic strains at age 1 week and at ages 1, 3, 4, and 6 months. The exploratory objectives included (1) hospitalization rates until age 6 months, (2) nasopharyngeal carriage of *Streptococcus pneumoniae* and other respiratory tract bacteria at age 2 weeks and at ages 1 and 4 months, (3) immunoglobulin G (IgG) antibody responses to childhood vaccination (PCV13; diphtheria, tetanus, and whole-cell pertussis [DTwP]; *Hemophilus influenzae* type b [Hib]; and hepatitis B), (4) the kinetics of probiotic colonization at various time points over the first 6 months of life, and (5) maternal carriage and colonization by probiotic species and pathogens at the time of delivery.

### Ethical Considerations

Ethics approval was obtained from the University of Western Australia (2018/RA/1/2301/4) and the PNGIMR Institutional Review Board (1809) in 2018 and the PNG Medical Research Advisory Committee (18.20) in 2019. The study has been registered as a clinical trial in PNG under the National Department of Health Pharmaceutical Standards Branch (CT04 MRAC18.20) and has also been registered with the Australian New Zealand Trials Registry (ACTRN12620001369910).

Before and throughout the study, clinic and field staff conducted awareness and information sessions within communities in and near Goroka, the Goroka Provincial Hospital prenatal clinic, and rural health centers. Formal letters and information about the study were also sent to the Eastern Highlands Provincial Health Authority and senior management of Goroka Provincial Hospital, from whom approval was obtained before study commencement.

Healthy, pregnant women late in the third trimester were approached in the prenatal clinic at Goroka Provincial Hospital or in their local communities by a study health extension officer (HEO) or nurse. The women were given information about the study and given the opportunity to discuss participation with their families. Women who assented to be part of the study were approached within 72 hours of giving birth at the Goroka Provincial Hospital labor ward or postnatal clinic. Their consent was confirmed, and if all inclusion criteria were met, the infant was enrolled in the study. Written parental consent was obtained before enrollment.

The study was conducted according to the Declaration of Helsinki, the International Conference on Harmonisation Good Clinical Practice, and local ethical guidelines. All participants were assigned a unique study number that was detached from their personal information. Personal data were accessible only to key investigators and were not linked with samples for processing. All data were therefore anonymized. Hard copies of participant information containing identifiable details were securely stored in locked offices. At the end of March 2023, due to higher-than-anticipated loss to follow-up because study infants’ families left the study area, largely owing to the COVID-19 pandemic, ethics approval was obtained to recruit additional participants.

### Inclusion and Exclusion Criteria

The inclusion and exclusion criteria are presented in Textbox 1.

If the mother of an already enrolled child developed fever or abdominal tenderness within 2 days of giving birth, the child remained eligible to stay in the study as long as the child was clinically well.

Infants were deemed eligible to participate and to not have any congenital abnormalities based on clinical assessments conducted routinely by hospital pediatricians on newborns delivered at Goroka Provincial Hospital and by a study HEO.

**Inclusion and exclusion criteria.**

**Inclusion criteria**

- Infant birth weight ≥1.5 kg
- Infant aged <72 h
- Infant feeding orally within the first 72 h of life
- Mother and infant residing within 60-min driving distance from the Papua New Guinea Institute of Medical Research in Goroka
- Family intending to remain in the study area for at least 6 mo (duration of the study)
- Parental written informed consent obtained

**Exclusion criteria**

- Mother HIV positive or of unknown status
- Mother with foul-smelling amniotic fluid
- Heavy meconium staining of amniotic fluid
- Mother with fever (>38 °C) within −2 or +2 d of delivery
- Mother with abdominal tenderness within 2 d of delivery
- Parents unable or unwilling to provide consent
- Infant receiving antibiotics between birth and screening (within the first 72 h of life)
- Infant with signs or symptoms of suspected infection at the time of screening
- Infant with a major congenital abnormality
- Infant with an Apgar score of <8

### Study Interventions

The synbiotic products and placebo were manufactured by DuPont Nutrition and Biosciences, now part of International Flavors and Fragrances Inc (IFF), and supplied in September 2020 before the study commenced, in quantities sufficient for the full target cohort. An additional 10% was provided to allow for repeat doses (eg, in cases of vomiting after administration) and for any additional enrollments to account for anticipated loss to follow-up. A full replacement batch was supplied by IFF in November 2022 due to the expiry of the initial batch before recruitment had been completed. The intervention products were shipped at room temperature and stored at the PNGIMR at 4 °C until use.

Two probiotic supplements—single-strain *L plantarum* and *B longum* subspecies *infantis—* compared to placebo were used in this study. These 2 probiotic supplements were prepared as synbiotics and supplied, as with the placebo, lyophilized in glass vials. Each vial contained a single dose of one of the following: (1) *L plantarum* (ATCC 202195; 1.0×10^9^ colony-forming units) + 150 mg of fructo-oligosaccharide, with 100 mg potato maltodextrin as excipient (matching the preparation used in the Indian trial [23]); (2) *B longum* subspecies * infantis* (Bi-26; at least 1×10^9^ colony-forming units) + 1.2 g of the human milk oligosaccharide 2′-fucosyllactose, with 100 mg potato maltodextrin as excipient; or (3) placebo (100 mg potato maltodextrin).

### Randomization

A computer-generated randomization list was maintained by DuPont Nutrition and Biosciences (now part of IFF) such that the research investigators, research and laboratory staff, and study participants remained blinded to the intervention arms. A single randomization code (sequential numbers 1-215) was assigned to each carton of intervention product, which contained 7 vials—one for each day of administration (d 1-d 7)—and was allocated consecutively to each enrolled child. Each vial was labeled with the specific trial randomization code and the corresponding day of administration. After the expiry of the initial batch, the replacement intervention products, also sequentially numbered, were used to continue enrollment beyond participant 215. The same blinding randomization procedures were followed to ensure allocation concealment and consistency throughout the trial.

### Intervention Administration, Vaccination, and Follow-Up

**Figure 1:**
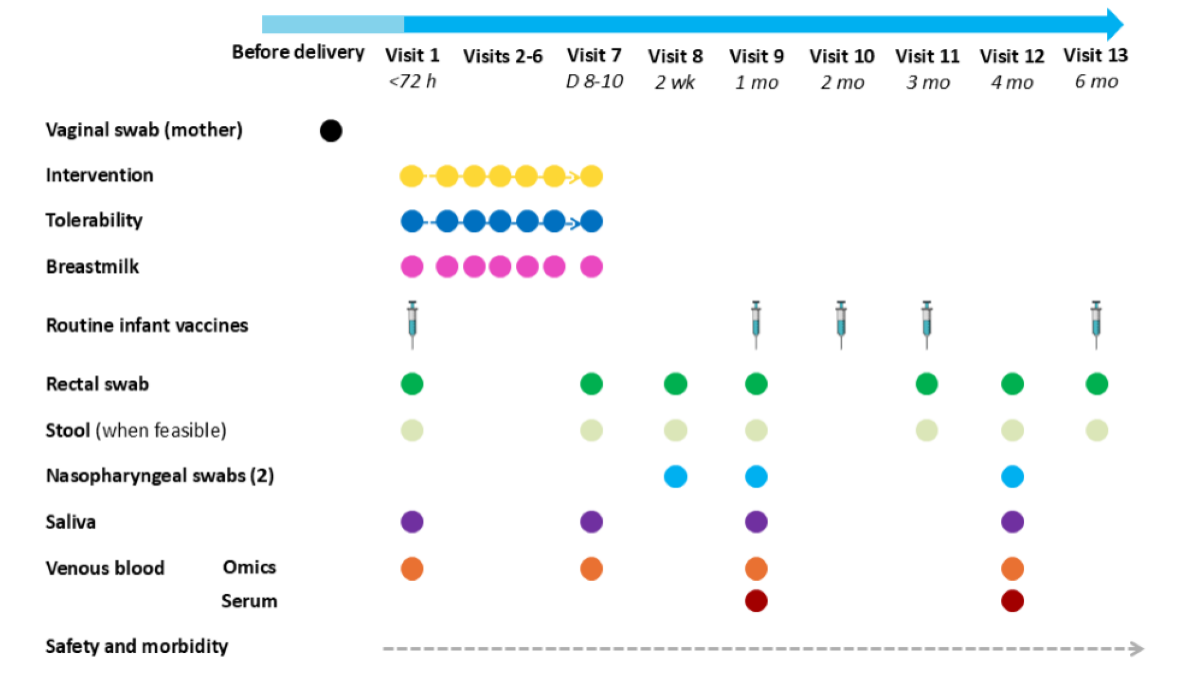
Schedule of synbiotic or placebo administration, routine infant vaccinations, and specimen collection throughout the study.

The schedule of synbiotic administration, routine infant vaccinations, and specimen collection throughout the study is shown in Figure 1. Just before administration, a single vial of intervention product corresponding to the appropriate day of administration (d 1-d 7) was diluted by adding 3 mL of either breast milk or sterile water directly to the vial, followed by inverting 5 times to mix and resuspend the contents. The resulting suspension was administered to the infant in oral drops using a Pasteur pipette, similar to the method used for oral polio vaccine. Before administration, infants were breastfed and burped. The intervention was administered by PNGIMR study staff (HEO or study nurse). When the intervention was administered at participants’ homes, the product vials were transported in small portable coolers with ice packs. The infant was monitored for 30 minutes after receiving the intervention. In cases of vomiting, the dose was repeated an hour later. A missed dose was defined as any scheduled daily dose (d 1-d 7) not administered on the same day, regardless of the reason, including participant unavailability, vomiting without readministration, or logistical barriers to follow-up. During the observation period, infants were taken to the PNGIMR clinic for examination and treatment (if the intervention had been administered elsewhere) and referred to Goroka Provincial Hospital when necessary. Obstetrics information and baseline characteristics were obtained on the day of enrollment. At each follow-up visit, the infant’s health was reviewed before any intervention administration, vaccination, or specimen collection. Weight and length measurements were recorded, and information on feeding practices was also collected at each visit.

Routine childhood vaccines, including the pentavalent vaccine (DTwP, hepatitis B, and Hib), PCV13, and oral polio vaccine (Figure 1) were obtained through the Eastern Highlands Provincial Health supply chain and administered by study HEOs or nurses at ages 1, 2, and 3 months as per PNG’s expanded program on immunization. Inactivated polio vaccine was also administered at age 3 months, and the first dose of the measles vaccine was administered at the completion of the study (6 mo). Birth doses of bacillus Calmette-Guérin (BCG) and hepatitis B vaccines were generally administered by hospital staff. In instances where this did not occur, study HEOs and nurses administered them at the time of enrollment. All vaccinations administered were recorded in the child’s health book held by the parent or guardian.

Follow-up visits were conducted at either the PNGIMR clinic or at the participant’s home. When blood collection was scheduled (at enrollment, on the day of the last intervention administration, and at ages 1 and 4 mo), participants were brought to the PNGIMR clinic for review and sample collection. All other follow-up visits were carried out by the clinical and field staff at the participants’ place of residence. Up to 3 home visit attempts were made for each participant follow-up before a child was withdrawn from the study after being deemed unlocatable. Children were also withdrawn from the study if information had been received that the family had permanently moved out of the study area, if parents or guardians withdrew consent, if a protocol violation occurred, or if the investigators determined that continued participation could adversely affect the child’s health.

### Morbidity Surveillance

All serious adverse events (SAEs) that occurred during the study period were recorded. The variability in diagnostic terms and a lack of gold standard case definitions for neonatal sepsis make it difficult to compare incidence, severity, etiology, or disease outcomes across studies and settings. Therefore, instead of sepsis, we examined “severe infection” as a clinical outcome, acknowledging that many infants will not manifest overt signs of sepsis and using the qualifier “severe” to indicate that localized infections without systemic features (eg, skin pustules) will be excluded. Severe clinical infection was defined as the presence of at least one of the following signs or symptoms to align with a similar study of synbiotics in Bangladesh [31]: (1) poor feeding (not sucking effectively or not sucking at all, as directly observed), (2) lethargy (movement only when stimulated or not moving at all, as directly observed), (3) convulsions (observed or strongly suspected by the clinician based on caregiver or community health worker report), (4) severe chest in-drawing (observed), (5) fever (axillary temperature of ≥38 °C), and (6) hypothermia (axillary temperature of <35.5 °C). In addition, any subsequent positive sterile site cultures or hospitalization with intention to treat with antibiotics for at least 5 days was considered indicative of severe clinical infection.

Throughout the study, the pediatric ward at Goroka Provincial Hospital was monitored daily for possible admissions of study infants. For any participant admitted, clinical information on health status, reason for admission, treatment, and discharge outcomes was recorded. In addition, parents or guardians were encouraged to bring their infants to the PNGIMR clinic for any illness experienced to enable SAEs to be recorded during this period. If clinically indicated, specimens were collected to aid in health assessment and diagnosis. Clinical samples included small volumes of blood (approximately 2 ml), stool, and a nasopharyngeal swab. Samples were processed at the PNGIMR laboratory, and the results were returned to the study clinicians for dissemination to the participant’s parent or guardian.

If a child was unwell during a scheduled study visit, routine procedures such as intervention administration and sample collection were still conducted unless contraindicated.

In addition, as in previous clinical trial studies led by the PNGIMR, study children’s family members who became unwell were also seen at the PNGIMR clinic as needed during the 6-month participation period.

### Specimen Collection

For the secondary and exploratory objectives of the study, a number of biological samples were collected at several time points throughout the study (Figure 1). Rectal swabs and stool samples were collected to assess intestinal colonization by probiotic species. Rectal swabs were collected at enrollment; on the day of the last intervention administration (age 7-10 d); and at ages 2 weeks, 1 month, 3 months, 4 months, and 6 months. For sample collection, the infant was carefully placed face down across the mother’s thighs. A FLOQSwab (Copan Diagnostics) was gently inserted approximately 2 cm into the anal canal, rotated slowly for 10 seconds, then removed and placed into a tube containing eNAT preservation and transport medium (Copan Diagnostics). The swab was broken at the breakpoint, leaving the tip in the tube, which was then sealed and mixed by inverting 10 times. Stool samples were collected in addition to rectal swabs if infants passed stool during the study visit to allow for sensitivity comparisons of probiotic strain detection in rectal swabs versus stool. Stool samples were taken from infants’ diapers using a scoop and placed into stool specimen containers for storage and transportation. Upon arrival at the PNGIMR laboratory, stool samples were transferred to cryovials by laboratory staff. Both rectal swabs and stool samples were then stored at −80 °C.

Nasopharyngeal swabs to assess bacterial carriage and load were collected at ages 2 weeks, 1 month, and 4 months using FLOQSwabs. At each time point, 2 swabs were collected per infant when possible. For each collection, a swab was inserted horizontally into the nostril to collect a superficial mucosal sample from the anterior nares, advancing gently until resistance was met. The swab was then rotated while counting to 5 (or for approximately 3 s) before being removed. One swab was placed in skim milk tryptone glucose glycerol broth (STGGB) for culturing of bacterial pathogens (priority), and the second was stored in PrimeStore molecular transport medium (Thermo Fisher Scientific) for deep sequencing. Both samples were stored at −80 °C.

Venous blood (maximum 2 mL at visits 1 and 2 and maximum 4 mL at visits 9 and 12) and saliva (visits 1, 7, 9, and 12) samples were obtained to assess systemic and mucosal vaccine responses and identify intervention-associated pathways or markers of immune maturation using systems biology approaches. Blood was collected using small 20-gauge butterfly needles and syringe techniques to reduce the risk of vein collapse. A maximum of 2 attempts were made to obtain a blood sample at each visit; if unsuccessful, no further attempts were made. For systems biology studies, we collected up to 2 mL of blood in heparin tubes. These samples were processed at the PNGIMR laboratory within 15 minutes of collection for RNA (transcriptomic analysis), plasma (proteomic and metabolomic analysis), flow (single cell immunophenotyping), and white blood cells (proteomic, metabolomic, and epigenetic analysis), following a protocol established by the Expanded Program on Immunization Consortium [32]. For serology, we collected up to 2 mL of blood in standard serum tubes, and the serum was separated and stored in cryovials by laboratory staff. Blood for serology was prioritized at ages 1 and 4 months over blood for systems biology. Saliva samples were collected from infants, ideally at least 30 minutes after being breastfed, using 4 to 6 surgical eye spears (Defries Industries) placed on the floor of the mouth to absorb saliva from the gums until saturated. The eye spears were then transported in 15 mL Falcon tubes (Corning Incorporated) to the PNGIMR laboratory where they were centrifuged at 10,000 g at 5 °C for 10 minutes. Saliva recovered after centrifugation was transferred to cryovials for storage. If no saliva was obtained after centrifugation, the sample was discarded. All blood products and saliva samples were initially stored at −80 °C at the PNGIMR before being shipped on dry ice to The Kids Research Institute Australia (formerly Telethon Kids Institute) in Perth, Western Australia, Australia.

Mothers were asked to self-collect vaginal swabs shortly after delivery using eNAT FLOQSwabs. These samples were used to collect information on the background prevalence of maternal vaginal carriage of probiotic species, in addition to pathogens associated with neonatal sepsis, such as group B *Streptococcus* and *Escherichia coli*, which may be vertically transmitted to the infant at the time of delivery. The swabs were stored at −80 °C.

Excess expressed breast milk was also collected at each intervention visit (visits 1-7) and stored at −80 °C at the PNGIMR for later analysis of protein (including antibodies) and carbohydrate content.

### Laboratory Procedures

#### Gut Colonization

Rectal swabs and stool samples will be tested for the presence of the probiotic species and strains used in this study using quantitative polymerase chain reaction (qPCR). Assays will be performed at the PNGIMR using an in-house method developed and optimized for local laboratory conditions. Briefly, total DNA will be extracted from approximately 200 mg of stool or 600 to 800 µL of rectal swab eNat preservation medium using the FastDNA Spin Kit for Soil (MP Biomedicals), incorporating a bead*-*beating step to enhance bacterial cell wall disruption.

Singleplex qPCR reactions will be used to detect *B longum* subspecies *infantis* and *L plantarum*, using the TaqMan Fast Advanced Master Mix (Thermo Fisher Scientific). Standard curves will be generated from DNA extracted from quantified cultures of *B longum* subspecies *infantis* and *L plantarum* grown either at the PNGIMR or at Federation University, Australia.

The primer and probe sequences to be used for *B longum* subspecies *infantis* are as follows:

- Bi26_Forward 5 - GTCACGATGTCTCCTTTGATATCA GCATG-3′
- Bi26_Reverse 5′- CCTTTTGCGTCTCCCCCG-3′
- Bi26_Probe 5 - FAM-TCATTCATTGTAGTGGCGATCAC CGTTACC-3′

The primer and probe sequences to be used for *L plantarum* are as follows:

- Lp_Forward 5′-ACAAATGGCAGGGCGAAAAATG-3′
- Lp_Reverse 5′-TTCTACATCCGGGCAAACCTTG-3′
- Lp_Probe 5′- FAM-ACGGAGCTTCTATGACGATGCTT TCA-3′

#### Pneumococcal Carriage

Nasopharyngeal swabs in STGGB will be cultured for the detection of respiratory bacterial pathogens, including *S pneumoniae*, *H influenzae*, *Moraxella catarrhalis*, and *Staphylococcus aureus*. Culturing and serotyping will be conducted at the PNGIMR using standard bacteriological culture, isolation, and identification methods, as previously described [27,33,34]. Briefly, 10 μL aliquots of STGGB samples will be inoculated onto horse blood agar, chocolate agar, gentamicin blood agar (5 μg/mL), and bacitracin chocolate agar (300 μg/mL) and incubated at 37 °C in 5% carbon dioxide for 18 to 24 hours. Bacterial growth will be semiquantified on the primary culture plates using a 7-point density scoring system (0-6), with scores of 1 to 3 indicating low-density colonization and 4 to 6 indicating high-density colonization.

Colony morphology and standard confirmatory tests will be used for bacterial identification. Presumptive *S pneumoniae* colonies will be confirmed by optochin sensitivity testing (≥14 mm inhibition zone) and, for optochin-resistant isolates, by bile solubility. *H influenzae* will be identified by growth characteristics on selective media and confirmed using X and V factor dependency tests. Up to 4 morphologically distinct pneumococci will be selected and serotyped using the Quellung reaction with commercially available antisera (Statens Serum Institut). *H influenzae* will be serotyped by slide agglutination (Remel). Antimicrobial susceptibility will be determined using the Kirby-Bauer disc diffusion method, with confirmation of suspected resistance by ETEST strips (bioMérieux) and interpretation according to Clinical and Laboratory Standards Institute breakpoints.

Samples will also be stored for future molecular analysis of total bacterial load and species-specific load using qPCR assays targeting bacterial DNA and pathogen-specific sequences.

#### Vaccine Antibody Responses (Serology)

Serum IgG antibody concentrations against PCV13 serotypes will be measured in blood samples collected at ages 1 and 4 months using a multiplexed immunofluorescent microsphere assay established at The Kids Research Institute Australia [35]. For each serotype, geometric mean concentrations and the proportion of children in each group with IgG levels of ≥0.35 μg/mL (considered the serological correlate of protection against invasive pneumococcal disease) will be calculated at both time points (ie, before immunization and 1 mo after the third PCV13 dose).

IgG responses to other childhood vaccines, including hepatitis B, DTwP, and Hib, will also be assessed using aliquots of the same serum samples. These will be analyzed using an in-house multiplexed immunoassay developed at The Kids Research Institute Australia laboratories [36]*.*

#### Systems Biology

Systems biology involves the application of different technologies that can detect all proteins, metabolites, gene transcripts (RNA), and cell types present in a blood sample taken at a specific time (proteomics, metabolomics, and transcriptomics). In this study, these technologies will be applied to identify specific molecular pathways and biomarkers that are modulated by the intervention and may be associated with improved immune development, such as enhanced vaccine responses, or greater protection against infectious diseases. Blood samples were collected and processed in accordance with protocols used and implemented previously by the Expanded Program on Immunization Consortium in PNG [32]. Processed blood samples will be stored at the PNGIMR until sufficient funding is secured to conduct these complex and high-cost analyses, at which point the samples will be shipped to the appropriate laboratories for analysis and data generation.

### Study Data and Management

All data collection forms were checked manually before entry into a password-protected electronic database management system (REDCap [Research Electronic Data Capture; Vanderbilt University]) housed at The Kids Research Institute Australia. Access to the deidentified database was accessible to authorized researchers and study personnel only at the PNGIMR and collaborating institutions. To ensure that any problems or queries were addressed as soon as possible, investigators in PNG and Australia attended fortnightly teleconferences.

### Data Safety Monitoring Board

A data safety monitoring board (DSMB) of independent clinicians from PNG and Australia was established. All SAEs were reported within 24 hours to an appointed Papua New Guinean safety monitor and the chair of the DSMB for review, followed by a final report detailing the outcome. Quarterly reports that included a summary of all SAEs, morbidities, and protocol violations as well as the status of enrollment and follow-up were prepared by the investigators and sent to the chair of the DSMB and distributed to other DSMB members. A teleconference with DSMB members and the study investigators was held every quarter.

### Sample Size Calculation

It was determined that a sample size of 50 participants per group would achieve 90% power to detect a difference in colonization rates of 30% in the probiotic group versus 5% in the placebo group, using a 2-tailed test of proportions with continuity correction at the 5% significance level. A final sample size of 65 participants per group was decided upon, taking into account a presumed 10% loss to follow-up and difficulties in timely biosample collection. On the basis of this sample size, it was estimated that the study would have 80% power to detect a 50% reduction in pneumococcal colonization rate in the probiotic group versus the placebo group at the 5% significance level.

### Statistical Analyses

The data will be cleaned before analysis. All data will be downloaded from REDCap and a backup copy archived before data cleaning. Missing data, outliers, and inconsistencies will be queried and verified against source documentation. Where data require changing, the database will be updated accordingly. All queries and corresponding amendments will be documented. Per-protocol and intention-to-treat analyses will be performed using available outcome data. The distribution of missing data across the treatment groups will be described, and reasons for missingness will be explored where possible to assess whether data are missing at random.

The primary objective is to determine the feasibility and safety of treatment. The number of solicited and unsolicited adverse events as well as the number of discontinued, missed, and completed doses will be summarized descriptively by treatment group. Pearson chi-square and Fisher exact tests will be used as appropriate to determine the significance of differences between groups.

The secondary objective is to measure gut colonization by probiotic strains on the last day of treatment (d 7-d 10) and at ages 1, 3, and 4 months. The prevalence of colonization at each time point will be summarized descriptively by treatment group. Colonization status, categorized as positive or negative, will be compared between treatment groups using the Pearson chi-square test, while continuous measures of colonization (ie, cycle threshold or relative fluorescence units) will be analyzed using 1-way ANOVA.

The exploratory objectives include measuring the hospitalization rates throughout the study; nasopharyngeal carriage of *S pneumoniae* and other respiratory bacteria; IgG antibody titers to PCV13, DTwP, Hib, and hepatitis B; the kinetics of probiotic gut colonization; and maternal carriage of probiotic species at the time of delivery.

Hospitalization rates will be reported descriptively per treatment group and compared using Pearson chi-square or Fisher exact tests as appropriate.

Nasopharyngeal carriage of *S pneumoniae* and other respiratory bacteria as well as gut colonization will be summarized descriptively at each corresponding time point. A Cox regression analysis will be performed, adjusting for birth weight, sex, previous antibiotic use, and breastfeeding status. Hazard ratios, along with their 95% CIs and *P* values, will be reported.

IgG antibody titers to PCV13, DTwP, Hib, and hepatitis B will be reported descriptively at baseline (before vaccination at age 1 mo) and at age 4 months (1 mo after the third dose). The differences between treatment groups will be estimated using linear regression, adjusting for birth weight, sex, breastfeeding status, and previous antibiotic use at each time point. The model will also adjust for maternal antibody titers to diphtheria, tetanus, and pertussis when comparing IgG titers to DTwP, using prevaccination infant titers as proxy measures. All IgG levels will be log transformed before analysis to meet the normality assumptions of linear regression analysis. The corresponding coefficients, along with their 95% CIs and *P* values, will be reported.

Maternal vaginal carriage of probiotic species will be reported descriptively and compared using the Pearson chi-square test.

Ad hoc analyses will include comparisons of IgG antibody levels, nasopharyngeal carriage of respiratory bacteria, SAEs, and other growth outcomes between participants colonized and those not colonized by *B longum* subspecies *infantis* or *L plantarum*. The Pearson chi-square test, the Fisher exact test, and regression modeling will be used as appropriate. The models will adjust for the variables specified earlier.

To ensure that normality assumptions are met before parametric testing, tests for normality (eg, the Shapiro-Wilk test) and a visual assessment of distribution using histograms will be carried out and the results reported. Where necessary (eg, antibody titers), the raw data will be log transformed to achieve a normal distribution.

The results of the analysis will be reported according to the CONSORT (Consolidated Standards of Reporting Trials) guidelines.

## Results

### Assent and Enrollment

**Figure 2:**
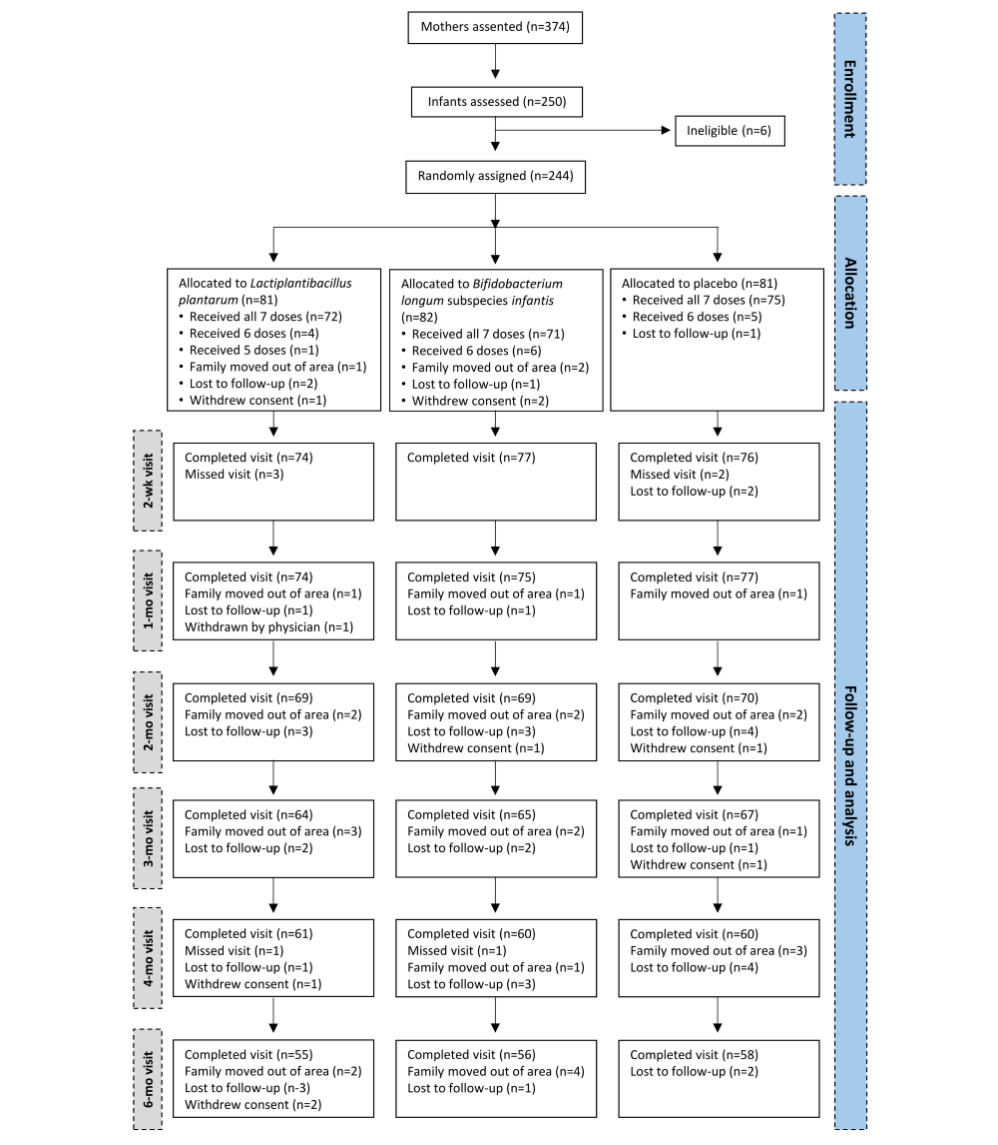
Flowchart of participant enrollment and visit completion. Of the 244 participants enrolled, 169 (69.3%) completed the study, with no substantial differences between treatment groups. “Family moved out of area,” “Lost to follow-up,” and “Withdrew consent” indicate study terminations, whereas “Missed visit” refers to skipped visits where participants remained enrolled.

**Figure 3:**
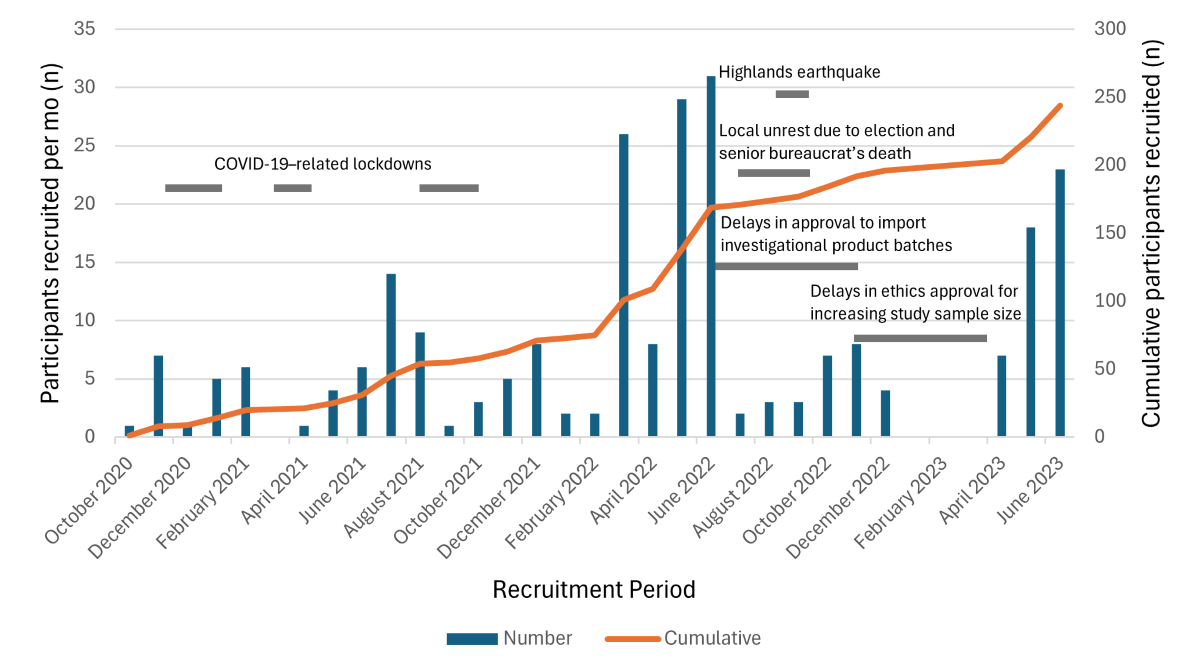
Participant recruitment patterns from October 2020 to June 2023. Overall, recruitment increased as the study progressed. Periods of disruption due to COVID-19–related lockdowns, national elections, civil unrest, delays in probiotic importation, and delays in ethics approval for study sample size expansion are reflected as lulls in recruitment.

A total of 244 children were enrolled between October 29, 2020, and June 30, 2023, and randomly assigned to receive *L plantarum* (ATCC 202195), *B longum* subspecies *infantis* (Bi-26), or placebo (maltodextrin). Figure 2 shows the number of women who provided assent, the number of infants randomly assigned, the number of children seen at subsequent visits, and the number of early terminations. Reasons for participant loss between maternal assent and infant screening were not systematically recorded; informal observations suggest that this was primarily due to early postnatal discharge, family disagreement after providing assent, or change of mind. In the first year of the study, only 63 (32.3%) of the target 195 infants were enrolled. This shortfall was largely due to the onset of the COVID-19 pandemic, which led to multiple government-imposed lockdowns and curfews that restricted field activity and hindered recruitment efforts. However, recruitment increased during the second year, and by the end of December 2022, the full cohort of 195 participants had been recruited. Nearly half of these participants (94/195, 48.2%) were enrolled between March and the end of June 2022 (Figure 3).

We had a higher-than-anticipated loss to follow-up because participants’ families left the study area during the study period, largely owing to the COVID-19 pandemic; hence, at the end of March 2023, we obtained ethics approval to recruit an additional 45 participants. This increased the target sample size from 195 to 240 (n=80 per treatment group). These additional participants were enrolled between April and June 2023 (Figure 3).

### Follow-Ups and Early Study Terminations

Of the 244 enrolled infants, 169 (69.3%) successfully completed the study by December 2023. Among the 75 early study terminations, 37 (49%) infants were lost to follow-up, 28 (37%) infants’ families moved out of the study area before study completion, and 9 (12%) infants’ parents withdrew consent. Moreover, of these 75 participants, 1 (1%) was withdrawn by the physician for medical reasons not related to the study intervention. The majority of these early terminations (59/75, 79%) occurred after the 1-month visit (Figure 2).

### Protocol Violations

There were no protocol violations; however, there was 1 incident of an accidental delivery of the study intervention to a nonstudy participant. This child was monitored over several days by study staff to ensure their well-being, and the parents were informed to contact a member of the study team in the event of any illness. No adverse events were subsequently reported for this child.

In addition, during July and August 2023, there was a shortage of routine vaccines in Goroka, which delayed some of the follow-up visits and specimen collections for 45 (18.4%) of the 244 study participants. Regarding the birth dose vaccines, 11 (4.5%) of the 244 participants did not receive hepatitis B, and 15 (6.1%) did not receive BCG; 5 (2%) received neither hepatitis B nor BCG, with 4 (80%) of these participants subsequently lost to follow-up before the 1-month visit. Of the 244 participants, 24 (9.8%) experienced delays in receiving BCG, with 14 (58%) of these participants receiving it 2 to 3 months after birth.

Among the routine monthly vaccination visits, delays were observed as follows: 7 (2.9%) of the 244 participants had their 1-month vaccines delayed, 11 (4.5%) had their 2-month vaccines delayed, and 4 (1.6%) had their 3-month vaccines delayed. For participants with a delayed monthly vaccination visit, the timing of all subsequent scheduled follow-up visits for sample collection and vaccination were readjusted accordingly; for instance, if a participant’s 1-month vaccines were delayed by 24 days, their 2- and 3-month vaccines were also delayed by 24 days. Of the 22 participants with delayed monthly vaccinations and sample collections, 3 (14%) were subsequently lost to follow-up: 1 (33%) before receiving their 1-month vaccinations and 2 (67%) before receiving their 3-month vaccinations. No participant remaining in the study experienced >1 additional delayed monthly vaccination.

### Feasibility

Of the 244 enrolled infants, 218 (89.3%) successfully completed the daily intervention from day 1 to day 7. Of the 26 newborns who did not complete daily treatment, 10 (38%; *L plantarum* group: n=4, 40%; *B longum* subspecies *infantis* group: n=5, 50%; and placebo group: n=1, 10%) were early study terminations, and 16 (62%; *L plantarum* group: n=5, 31%; *B longum* subspecies *infantis* group: n=6, 38%; and placebo group: n=5, 31%) missed ≥1 doses (n=15, 94% missed a single dose, and n=1, 6% missed 2 doses) but continued in the study. Among the 10 participants who exited the study early, in the *L plantarum* group, 2 (50%) of the 4 participants received only 1 dose, 1 (25%) received 3 doses, and 1 (25%) received 6 doses; in the *B longum* subspecies *infantis* group, 1 (20%) of the 5 participants received 2 doses, 1 (20%) received 4 doses, 1 (20%) received 5 doses, and 2 (40%) received 6 doses; and in the placebo group, the participant lost to follow-up (1/1, 100%) received only 1 dose.

### Specimen Collection

Specimen collection occurred in parallel with recruitment, from October 2020 to December 2023, with samples obtained during scheduled follow-up visits from birth through 6 months of age. Rectal swabs, nasopharyngeal swabs, saliva, and blood were successfully collected from >91% of the infants who were still enrolled in the study at the time of their follow-up visits (Table 1). Sufficient blood volumes were collected from 80.5% (182/226) and 80.9% (144/178) of the participants at the 2 time points where >1 analysis was proposed for the sample. Rectal swabs were collected from >99% of the participants at each of the 7 time points, except at the 3-month visit, where the collection rate was 91.3% (179/196). Collection of stool was low, with the majority of the samples (193/478, 40.4%) being obtained within the first week of the study. Two nasopharyngeal swabs were collected from >98% of the participants at each of the 3 time points. Of the 881 saliva sponge specimens collected, 183 (20.8%) failed to recover a viable sample for subsequent analyses after centrifugation and were discarded. Excess breast milk was successfully obtained from mothers for up to 7 days during the first week of the study in 75% (183/244) of the cases. However, only 17.6% (43/244) of vaginal swabs were obtained. Data analysis is ongoing and is expected to be completed by the end of 2025.

**Table 1:** Participant specimens collected at designated time points during the 6-month follow-up.

Sample types				Mothers, n (%)		Infant age, n (%)												
						<72 h (n=244)		8-10 d (n=234)		2 wk (n=227)		1 mo (n=226)		3 mo (n=196)		4 mo (n=178)		6 mo (n=169)
**Maternal**																		
	Vaginal swabs (n=244)			43 (17.6)		—^a^		—		—		—		—		—		—
	Breast milk (n=1708)			1281 (75)		—		—		—		—		—		—		—
**Infant**																		
	Rectal swabs			—		244 (100)		233 (99.6)		225 (99.1)		226 (100)		179 (91.3)		177 (99.4)		168 (99.4)
	Stool			—		89 (36)		104 (44.4)		27 (11.9)		28 (12.4)		22 (11.2)		10 (5.6)		8 (4.7)
	Nasopharyngeal swabs (×2)			—		—		—		448 (98.7)		451 (99.8)		—		356 (100)		—
	Saliva			—		244 (100)		233 (99.6)				226 (100)		—		178 (100)		—
	**Blood**																	
		Omics	—		244 (100)		232 (99.1)		—		182 (80.5)		—		144 (80.9)		—	
		Serology	—		—		—		—		225 (99.6)		—		175 (98.3)		—	

^a^Not applicable.

## Discussion

### Feasibility of Administering Probiotics to Infants in PNG

Evidence supporting the benefits of probiotics for infant health is scarce but warrants further exploration, given the health benefits of probiotic use in preterm neonates. A study conducted in India demonstrated reduced risk of NEC and mortality in children receiving probiotics early in life, highlighting their potential as an effective health intervention in neonates in LMICs [23]. Given the numerous variables that can influence probiotic efficacy—including strain specificity, dose, and formulation; the timing and duration of administration; host factors such as age and nutritional status; and differences in microbiota composition—additional studies are required to determine the optimal conditions under which such interventions can be safely and effectively adopted in different populations. This pilot study aimed to assess the feasibility and safety of administering probiotic or placebo supplementation to neonates in PNG for 7 consecutive days—marking the first such study in this population.

Our study is one of very few examining the effects of probiotics in healthy infants in a community setting within an LMIC, and particularly in exploring immunological outcomes. As highlighted by a systematic review conducted by Imdad et al [37], research on probiotics in community settings in LMICs is lacking. By addressing this gap, we have demonstrated both successes and challenges in participant recruitment and fieldwork, with laboratory outcomes to follow (once analyses are completed).

We successfully completed participant recruitment, probiotic administration, and specimen collection in a challenging and labor-intensive research environment. Crucial to this success was maintaining a strong rapport with hospital staff, particularly those in the labor ward, which was a testament to the commitment of the study team. Daily home visits were conducted by study staff for synbiotic administration, resulting in 89.3% (218/244) of the study infants receiving all doses, and >95% (233/244) receiving at least 6 of 7 doses. Slightly more than two-thirds of the recruited participants (169/244, 69.3%) were followed through to completion, despite the challenges posed by the COVID-19 pandemic. These results demonstrate the feasibility of implementing such an intervention study in a community setting in PNG.

### Specimen Collection: Successes and Challenges

This study ambitiously sought to collect up to 27 specimens per infant across 13 visits. For rectal swabs, nasopharyngeal swabs, saliva, and blood for serology, the rate of collection was >98% at all time point–specimen combinations except for 1 (at the 3-mo collection point, 179/196, 91% of the participants provided a rectal swab; Table 1). Blood for serology (vaccine immunogenicity) was prioritized over blood for potential omics studies at the 1- and 4-month collection points; thus, the collection rate was lower for omics research at these time points (approximately 80%). Stool samples (collected in addition to rectal swabs) were sought “when feasible” and had a low collection rate, particularly at ages 4 and 6 months when only approximately 5% of participants (10/178 at 4 mo; 8/169 at 6 mo) provided a stool sample. Likely reasons include timing (the infant did not pass stool during the visit) and perhaps a cultural reluctance to provide stool samples in this setting [38].

Viable saliva was not recoverable from 20.8% (183/881) of the collected samples. Almost half of these (74/183, 40.4%) were from samples collected within 8 to 10 days of birth, and therefore likely a reflection of age and the inability to produce sufficient quantities of saliva. The high overall collection rates demonstrated the feasibility of intensive specimen collection in this setting. Future studies will streamline specimen collection based on the findings from this study.

In addition to specimens from infants, we also sought breast milk samples during the first 7 days of participation and a vaginal swab self-collected from mothers of infant participants before giving birth. Breast milk collection was successful, with 75% (1281/1708) of potential samples being obtained. However, <20% (43/244) of the mothers provided a vaginal swab, likely due to the allowable time for recruitment and enrollment in this study of up to 72 hours after delivery. While not critical to this study’s outcomes, vaginal microbiota could be relevant in future research on infant gut colonization.

### Logistical and Operational Barriers

Implementing this study in PNG required overcoming significant logistical challenges, including impassable roads, frequent road tolls, and maintenance of study vehicles. Due to the intensive nature of the follow-up visits, study staff had to remain flexible, and it was often the case that a different member of the team attended different follow-up visits for a particular participant. This was the reason for the accidental probiotic delivery to a nonstudy infant, but this incident also reflected the high level of trust the community places in the PNGIMR. On this particular visit, the study participant was not at their place of residence, and another infant was present. The staff member administering the intervention had not attended this participant previously, and the parent or guardian of the infant who was present gave no indication that the child was not part of the study and allowed the intervention to be administered. It is likely that there was a misunderstanding on the part of the parent or guardian, and the probiotic was perceived as a medicine and of benefit to the infant.

Additional logistical hurdles included a vaccine supply shortage in the EHP in 2023. Study participants received their routine vaccinations from study nurses using vaccines acquired through the government medical supply chain. This supply was temporarily disrupted during July and August 2023, delaying vaccine administration for 45 (18.4%) of the 244 participants. Furthermore, we experienced difficulties in importing a second batch of synbiotics into PNG due to delays in obtaining approvals from the relevant government organizations. Subsequent delays in obtaining ethics approval to expand the study size by >10% contributed to a lull in recruitment from January to April 2023 (Figure 3).

Adding to the complexity of this study and further compounding the challenges, recruitment occurred during the COVID-19 pandemic, which began in PNG in late October 2020. Although case numbers remained low until March 2021, the subsequent approximately 12 months were marked by high transmission, including the emergence of the delta variant in September 2021 and the first reported case of omicron in December 2021. The pandemic impacted study recruitment in numerous ways. First and foremost, government-mandated lockdowns and curfews limited field activities, including participant recruitment, particularly during periods of high transmission. Second, study staff experienced sick leave as well as family care responsibilities related to COVID-19 prevention and treatment. In addition, some study infants’ families relocated during the pandemic, resulting in loss to follow-up (65/244, 26.6%) or participant withdrawal (9/244, 3.7%).

In PNG, the concept of probiotics and “good germs” is not well understood, and the study team had to invest considerable time and resources to promote awareness in the community before and throughout the study, which likely contributed to the initially slow pace of recruitment. These challenges were compounded by the rollout of the COVID-19 vaccine. There was considerable hesitancy around the vaccine in PNG, resulting in very low vaccine uptake. The first COVID-19 vaccine was introduced in the country at the end of March 2021. As of the end of 2023 (the latest data available), <5% of the population had received ≥1 doses [39]. At the time of the vaccine rollout in the EHP, commencing in May 2021, there was confusion in some study communities: the probiotics were mistakenly believed to be COVID-19 vaccines, raising concerns that we were testing the vaccines on the infants.

In PNG, as in many countries, periods of political and civil unrest can occur. Elections can be highly contested; thus, conducting fieldwork in the lead-up to, during, and shortly after elections can be dangerous. During the national elections in mid-2022, fieldwork was considerably reduced. Not long after the election in 2022 (but not related to it), in September, the death of a senior bureaucrat in Goroka triggered violence and arson attacks, causing an estimated 3000 people to flee their homes. This incident also led to a pause in recruitment and follow-up. Around the same time, an earthquake struck the PNG highlands, damaging infrastructure, including roads; and contributing to further, albeit minor, delays in recruitment and follow-up.

### Implications for Future Research

While intervention studies can be challenging to implement in resource-constrained settings, it is in these settings that health interventions are most needed to improve health outcomes. Through our pilot study, we have demonstrated the feasibility of administering probiotics to infants in PNG in a clinical trial environment, supporting the implementation of a larger randomized controlled trial to study clinical impact.

The aforementioned challenges no doubt contributed to the early study terminations (75/244, 30.7%). However, of the 244 participants, 218 (89.3%) completed their 7-day course of probiotic or placebo. Of these 218 participants, 15 (6.9%) missed only 1 of 7 doses (n=233 , >95% had at least 6 of 7 doses). The comparatively high number of participants completing ≥6 doses provides some evidence that such an intervention could be feasible and be met with public acceptance, provided that sufficient education and dissemination of information accompanied the intervention.

## Abbreviations

ATCC: American Type Culture Collection
BCG: bacillus Calmette-Guérin
CONSORT: Consolidated Standards of Reporting Trials
DSMB: data safety monitoring board
DTwP: diphtheria, tetanus, and whole-cell pertussis
EHP: Eastern Highlands Province
HEO: health extension officer
Hib: Hemophilus influenzae type b
IgG: immunoglobulin G
LMIC: low- or middle-income country
LRTI: lower respiratory tract infection
NEC: necrotizing enterocolitis
PCV13: 13-valent pneumococcal conjugate vaccine
PNG: Papua New Guinea
PNGIMR: Papua New Guinea Institute of Medical Research
qPCR: quantitative polymerase chain reaction
REDCap: Research Electronic Data Capture
SAE: serious adverse event
STGGB: skim milk tryptone glucose glycerol broth

## Data Availability

The datasets generated and analyzed during this study will be available from the corresponding author on reasonable request once analysis has been completed and results published.

## References

[1] Levels and trends in child mortality. United Nations.

[2] Newborn mortality. World Health Organization.

[3] Araya M, Morelli L, Reid G, Sanders M, Stanton C, Pineiro M, Ben EP (2002). Joint FAO/WHO Working Group report on drafting guidelines for the evaluation of probiotics in food. Food and Agriculture Organization of the United Nations & WHO.

[4] Hill C, Guarner F, Reid G, Gibson GR, Merenstein DJ, Pot B, Morelli L, Canani RB, Flint HJ, Salminen S, Calder PC, Sanders ME (2014). Expert consensus document. The international scientific association for probiotics and prebiotics consensus statement on the scope and appropriate use of the term probiotic. Nat Rev Gastroenterol Hepatol.

[5] Guarner F, Sanders ME, Szajewska H, Cohen H, Eliakim R, Herrera-deGuise C, Karakan T, Merenstein D, Piscoya A, Ramakrishna B, Salminen S, Melberg J (2024). World Gastroenterology Organisation global guidelines: probiotics and prebiotics. J Clin Gastroenterol.

[6] Pandey KR, Naik SR, Vakil BV (2015). Probiotics, prebiotics and synbiotics- a review. J Food Sci Technol.

[7] Yadav MK, Kumari I, Singh B, Sharma KK, Tiwari SK (2022). Probiotics, prebiotics and synbiotics: Safe options for next-generation therapeutics. Appl Microbiol Biotechnol.

[8] Markowiak P, Śliżewska K (2017). Effects of probiotics, prebiotics, and synbiotics on human health. Nutrients.

[9] Mahboobi S, Rahimi F, Jafarnejad S (2018). Effects of prebiotic and synbiotic supplementation on glycaemia and lipid profile in type 2 diabetes: a meta-analysis of randomized controlled trials. Adv Pharm Bull.

[10] Ford AC, Harris LA, Lacy BE, Quigley EM, Moayyedi P (2018). Systematic review with meta-analysis: the efficacy of prebiotics, probiotics, synbiotics and antibiotics in irritable bowel syndrome. Aliment Pharmacol Ther.

[11] Chan CK, Tao J, Chan OS, Li HB, Pang H (2020). Preventing respiratory tract infections by synbiotic interventions: a systematic review and meta-analysis of randomized controlled trials. Adv Nutr.

[12] Pham VT, Mohajeri MH (2018). The application of in vitro human intestinal models on the screening and development of pre- and probiotics. Benef Microbes.

[13] Aabed K, Shafi Bhat R, Moubayed N, Al-Mutiri M, Al-Marshoud M, Al-Qahtani A, Ansary A (2019). Ameliorative effect of probiotics (Lactobacillus paracaseii and Protexin®) and prebiotics (propolis and bee pollen) on clindamycin and propionic acid-induced oxidative stress and altered gut microbiota in a rodent model of autism. Cell Mol Biol (Noisy-le-grand).

[14] Shelby RD, Janzow GE, Mashburn-Warren L, Galley J, Tengberg N, Navarro J, Conces M, Bailey MT, Goodman SD, Besner GE (2020). A novel probiotic therapeutic in a murine model of colitis. Gut Microbes.

[15] AlFaleh K, Anabrees J (2014). Probiotics for prevention of necrotizing enterocolitis in preterm infants. Cochrane Database Syst Rev.

[16] Aceti A, Gori D, Barone G, Callegari ML, Di Mauro A, Fantini MP, Indrio F, Maggio L, Meneghin F, Morelli L, Zuccotti G, Corvaglia L, Italian Society of Neonatology (2015). Probiotics for prevention of necrotizing enterocolitis in preterm infants: systematic review and meta-analysis. Ital J Pediatr.

[17] Chi C, Li C, Buys N, Wang W, Yin C, Sun J (2021). Effects of probiotics in preterm infants: a network meta-analysis. Pediatrics.

[18] Chi C, Buys N, Li C, Sun J, Yin C (2019). Effects of prebiotics on sepsis, necrotizing enterocolitis, mortality, feeding intolerance, time to full enteral feeding, length of hospital stay, and stool frequency in preterm infants: a meta-analysis. Eur J Clin Nutr.

[19] Agarwal R, Sharma N, Chaudhry R, Deorari A, Paul VK, Gewolb IH, Panigrahi P (2003). Effects of oral Lactobacillus GG on enteric microflora in low-birth-weight neonates. J Pediatr Gastroenterol Nutr.

[20] Balasubramanian H, Ananthan A, Rao S, Patole S (2020). Probiotics for preterm infants in India - systematic review and meta-analysis of randomized controlled trials. Indian J Pediatr.

[21] Deshpande G, Jape G, Rao S, Patole S (2017). Benefits of probiotics in preterm neonates in low-income and medium-income countries: a systematic review of randomised controlled trials. BMJ Open.

[22] FDA raises concerns about probiotic products sold for use in hospitalized preterm infants. U.S. Food and Drug Administration.

[23] Panigrahi P, Parida S, Nanda NC, Satpathy R, Pradhan L, Chandel DS, Baccaglini L, Mohapatra A, Mohapatra SS, Misra PR, Chaudhry R, Chen HH, Johnson JA, Morris JG, Paneth N, Gewolb IH (2017). A randomized synbiotic trial to prevent sepsis among infants in rural India. Nature.

[24] Papua New Guinea country office annual report 2022. United Nations Children's Fund.

[25] End preventable deaths of newborns and children under 5 years of age. World Health Organization.

[26] Annual child morbidity and mortality reports. The Paediatric Society of Papua New Guinea.

[27] Aho C, Michael A, Yoannes M, Greenhill A, Jacoby P, Reeder J, Pomat W, Saleu G, Namuigi P, Phuanukoonnon S, Smith-Vaughan H, Leach AJ, Richmond P, Lehmann D, Neonatal Pneumococcal Conjugate Vaccine Trial Study Team (2016). Limited impact of neonatal or early infant schedules of 7-valent pneumococcal conjugate vaccination on nasopharyngeal carriage of in Papua New Guinean children: a randomized controlled trial. Vaccine Rep.

[28] Britton KJ, Pickering JL, Pomat WS, de Gier C, Nation ML, Pell CL, Granland CM, Solomon V, Ford RL, Greenhill A, Hinds J, Moore HC, Richmond PC, Blyth CC, Lehmann D, Satzke C, Kirkham LS, 10v13vPCV trial team (2021). Lack of effectiveness of 13-valent pneumococcal conjugate vaccination against pneumococcal carriage density in Papua New Guinean infants. Vaccine.

[29] World Bank open data. The World Bank.

[30] Population estimates 2021. National Statistical Office of Papua New Guinea.

[31] Pell LG, Qamar H, Bassani DG, Heasley C, Funk C, Chen CY, Shawon J, O'Callaghan KM, Pullenayegum E, Hamer D, Haque R, Kabir M, Ahmed T, O'Kelly C, Hossain M, Khan A, Loutet M, Islam M, Morris S, Shah P, Sherman P, Sultana S, Mahmud A, Saha S, Sarker S, Roth D (2025). Neonatal administration of ATCC 202195 with or without fructooligosaccharide in Bangladesh: a placebo-controlled randomized trial. mSphere.

[32] Lee AH, Shannon CP, Amenyogbe N, Bennike TB, Diray-Arce J, Idoko OT, Gill EE, Ben-Othman R, Pomat WS, van Haren SD, Cao KL, Cox M, Darboe A, Falsafi R, Ferrari D, Harbeson DJ, He D, Bing C, Hinshaw SJ, Ndure J, Njie-Jobe J, Pettengill MA, Richmond PC, Ford R, Saleu G, Masiria G, Matlam JP, Kirarock W, Roberts E, Malek M, Sanchez-Schmitz G, Singh A, Angelidou A, Smolen KK, Brinkman RR, Ozonoff Al, Hancock RE, van den Biggelaar AH, Steen H, Tebbutt SJ, Kampmann B, Levy O, Kollmann TR, EPIC Consortium (2019). Dynamic molecular changes during the first week of human life follow a robust developmental trajectory. Nat Commun.

[33] Satzke C, Turner P, Virolainen-Julkunen A, Adrian PV, Antonio M, Hare KM, Henao-Restrepo AM, Leach AJ, Klugman KP, Porter BD, Sá-Leão R, Scott JA, Nohynek H, O'Brien KL, WHO Pneumococcal Carriage Working Group (2013). Standard method for detecting upper respiratory carriage of Streptococcus pneumoniae: updated recommendations from the World Health Organization Pneumococcal Carriage Working Group. Vaccine.

[34] Orami T, Aho C, Ford RL, Pomat WS, Greenhill A, Kirkham L, Masiria G, Nivio B, Britton KJ, Jacoby P, Richmond PC, van den Biggelaar AH, Lehmann D (2023). Pneumococcal carriage, serotype distribution, and antimicrobial susceptibility in Papua New Guinean children vaccinated with PCV10 or PCV13 in a head-to-head trial. Vaccine.

[35] Corscadden KJ, Kirkham LA, Thornton RB, Vijayasekaran S, Coates HL, Richmond PC, Wiertsema SP (2013). High pneumococcal serotype specific IgG, IgG1 and IgG2 levels in serum and the middle ear of children with recurrent acute otitis media receiving ventilation tubes. Vaccine.

[36] McAlister SM, van den Biggelaar AH, Thornton RB, Richmond PC (2021). Optimising a 6-plex tetanus-diphtheria-pertussis fluorescent bead-based immunoassay. MethodsX.

[37] Imdad A, Rehman F, Davis E, Ranjit D, Surin G, Attia S, Lawler S, Smith A, Bhutta Z (2021). Effects of neonatal nutrition interventions on neonatal mortality and child health and development outcomes: a systematic review. Campbell Syst Rev.

[38] Abdad MY, Soli KW, Pham B, Bande G, Maure T, Jonduo M, Kisa D, Rai G, Phuanukoonnon S, Siba PM, Horwood PF, Greenhill AR (2020). Diarrhoeal disease surveillance in Papua New Guinea: findings and challenges. Western Pac Surveill Response J.

[39] COVID-19 vaccines: WHO COVID-19 dashboard. World Health Organization.

